# Biopolymeric Delivery Systems for Cosmetic Applications Using *Chlorella vulgaris* Algae and Tea Tree Essential Oil

**DOI:** 10.3390/polym12112689

**Published:** 2020-11-14

**Authors:** Flávia P. Morais, Rogério M. S. Simões, Joana M. R. Curto

**Affiliations:** 1 Fiber Materials and Environmental Technologies Research Unit (FibEnTech-UBI), University of Beira Interior, Rua Marquês d’Ávila e Bolama, 6201-001 Covilhã, Portugal; rmss@ubi.pt; 2 Chemical Process Engineering and Forest Products Research Centre (CIEPQPF), University of Coimbra, R. Sílvio Lima, Polo II, 3004-531 Coimbra, Portugal

**Keywords:** biopolymers, *Chlorella vulgaris*, delivery systems, dermic and cosmetic applications, nanocellulose, porosity, tea tree oil

## Abstract

Cosmetic products in which all the skincare compounds are biomolecules, biocompatible and biodegradable constitute a request of an educated consumer corresponding to a premium cosmetic segment. For this purpose, a cellulose-based delivery system was developed to retain biomolecules for dermic applications. The 3D matrix was built with microfibrillated cellulose, nanofibrillated cellulose and carboxymethylcellulose combined with a crosslinking agent, the alginate, to obtain a 3D matrix capable of retaining and releasing bioactive components of microalgae *Chlorella vulgaris* and tea tree essential oil. The porosity and pore dimensions and uniformity of this support matrix were optimized using 3D computational tools. The structures of the biopolymers were characterized using SEM, EDX, FTIR-ATR and DSC techniques. The essential oil and the microalgae components were successfully incorporated in a 3D stable matrix. The results indicate that the polymeric matrix retains and releases the essential oil biomolecules in a controlled way, when compared with tea tree essential oil, which is vaporized from 25 °C to 38 °C, without this 3D polymeric matrix. The microalgae and cellulose-based delivery system proved to be an interesting option for dermic and cosmetic applications because the exposure time of the therapeutic biomolecules was improved, and this factor consists of a competitive benefit for dermic systems.

## 1. Introduction

Microalgae, an alternative raw material for biological economies, can convert light, water and carbon dioxide into biomass to provide useful biomolecules for various applications. An example of microalgae is the species *Chlorella vulgaris*. It is a species of green eukaryotic photolithoautotroph unicellular organism with high protein content, currently one of the most widely studied algae [1]. Recently, the search for cosmetic and pharmaceutical products of natural sources has led to microalgae research as a source of some value-added products of interest, such as carotenoids, proteins and vitamins [2]. Carotenoids can exhibit skin’s anti-aging and regenerative properties by softening their surface, particularly scars, and can significantly reduce the appearance of stretch marks and are particularly useful in treating any skin tissue that is physically harmed [3,4,5,6,7,8].

Skin aging is associated with structural and functional changes, together with greater skin vulnerability and dryness. Dry skin is the most common symptom of dermatological diseases [9]. The use of cosmetics can prevent dermatological diseases from dehydration. Microalgae components and essential oils can slow the loss of water due to the ability to occlude. Wetting substances have the capacity to attract water promoting hydration [10]. With the increase in life expectancy, the demand for effective cosmetic products for the prevention and treatment of different skin changes due to aging has been a constant. An example of an essential oil with good cosmetic properties is tea tree oil. It is an essential oil of the species *Melaleuca alternifolia*, composed of several components, with terpinen-4-ol considered as the active substance [11]. In addition to its antibacterial properties, tea tree essential oil relieves signs of acne, psoriasis and superficial burns and stimulates cut skin to heal while protecting it from infections [12,13,14].

The delivery systems development that controls the release rate of biomolecules in specific treatments is the key factor of research in the cosmetic and pharmaceutical fields. Over the years, the use of biopolymers in the production of delivery systems has been increasing [15,16,17,18]. By incorporating biomolecules into polymeric fibers, systemic and local/regional therapy can be provided compared to other systems (nanoparticles, nanocapsules, micellar systems, for example). This is because these latter systems exhibit intrinsic fluidity [19], which compromises their permanence and localization in a specific area of the body, consequently hindering localized therapy. Advantages such as low incidence of side effects, therapeutic efficacy [19] and biocompatibility [20] ensure the usefulness of polymeric materials in delivery systems. Alginate and cellulose derivatives, such as microfibrillated cellulose (MFC), nanofibrillated cellulose (NFC) and carboxymethylcellulose (CMC), are examples of natural polymeric materials that can be used in new and advanced applications [21,22,23].

Several strategies have been applied for the use of microalgae in targeted therapy delivery systems. Studies have reported that microalgae can be used as natural swimmers of delivery agents to transport therapeutic molecules within the body to hard-to-reach locals [24], as well as to provide cancer cells with chemotherapeutic drugs through their nanoporous biosilica [25]. However, to the best of our knowledge, the design of a delivery system containing a cellulose-based porous 3D matrix that retains microalgae and essential oils for dermic and cosmetic applications has not been thoroughly investigated.

The objectives of this study were to develop a delivery system based on cellulose biopolymers capable of releasing the bioactive components of microalgae *Chlorella vulgaris* and tea tree essential oil. Techniques such as optical microscopy, scanning electron microscopy (SEM), energy-dispersive X-ray spectroscopy (EDX), Fourier-transform infrared spectroscopy with attenuated total reflectance (FTIR-ATR) and differential scanning calorimetry (DSC) were used to characterize the biopolymeric materials used in the delivery system. An approach of combining experimental characterization and computational modeling was also carried out to optimize the porosity of biopolymeric delivery systems.

## 2. Materials and Methods

### 2.1. Materials

Bleached kraft *Eucalyptus globulus* pulp, air dried, was obtained from an industrial pulp mill and hydrated at FibEnTech Research Unit (University of Beira Interior, Covilhã, Portugal) using distilled water. CMC sodium salt, high viscosity (1500–3000) cP in 1% H_2_O (25 °C) was purchased from Sigma Aldrich (Darmstadt, Germany). Alginate sodium salt, with high viscosity, was acquired from BDH Chemicals Ltd. (London, UK). Tea tree essential oil was purchased at a local store. The microalgae *Chlorella vulgaris* was obtained from Aqualgae (Viana do Castelo, Portugal). All other chemicals and reagents used in the study were analytical grade.

### 2.2. Microalgae Production

For the growth of microalgae *Chlorella vulgaris*, GoldMedium Fresh-Water Species (GM-FWS) medium was used, prepared from different nutrients. The microalgae were grown in 500 mL gas washing bottles in shelves for microalgae growth, comprising an air pump model V-60 (Hailea, Guangdong, China) and a control unit for the light-dark cycles (12:12) using four fluorescent cylindrical LED. The shelf also possesses three airflow meters and three flow lines of CO_2_, all of them with time-controlled electro-valves.

### 2.3. MFC and NFC Production

For MFC production, 30 g (o.d. base) of bleached *kraft Eucalyptus globulus* pulp was hydrated overnight at pH 12 and disintegrated according to ISO 5263/1 standard (30,000 revolutions). After this process, a suspension with 10% consistency was obtained. The pulp was beaten in a PFI mill at 9000 revolutions under a refining intensity of 3.33 N/mm, according to ISO 5264/2 standard.

For NFC production, the pulp beaten as above was treated chemically using TEMPO (2,2,6,6-tetramethylpiperidine-1-oxyl radical)-mediated oxidation in a 4 dm^3^ reactor with pH control. This treatment consists of cellulose fibers oxidation with the addition of NaClO solution to cellulose suspension, in the presence of catalytic amounts of TEMPO and NaBr dissolved at pH 10–11 and room temperature [26]. The final mechanical treatment of these fibers was accomplished with a high-pressure homogenizer at 500 bars and temperature ranging from 20 °C to 39 °C.

### 2.4. 3D Biopolymeric Matrix Formation

A 3D matrix was produced with the MFC and NFC suspensions and, as an additive, CMC at 0.01% (*m/v*), in a 2:2:1 ratio. The 3D polymeric matrix was produced using gentle filtration with a Büchner funnel, after mixing under controlled stirring, temperature, time and pressure.

### 2.5. Delivery System Production

Delivery systems were produced by mixing an alginate solution (2% (*m/v*)), as natural crosslinking polymer, MFC-NFC-CMC gel, as an internal 3D matrix, the *Chlorella vulgaris* algae solution (1.33 g/L), in a 10:5:1 ratio, and 2.5 mL of tea tree essential oil was added to this mixture. This suspension was stirred for 30 min and then added dropwise to a CaCl_2_ solution to produce the reticulation. After 24 h of hardening, the systems were filtered, rinsed and washed with distilled water, with subsequent drying. From this methodology it was possible to obtain delivery systems, approximately, with the microalgae at a concentration of 1 × 10^−3^ g/mL and 1 mL of essential oil, an amount of encapsulation in the DDS in order to be efficient.

Another similar system was produced under the same conditions but without the seaweed solution, to understand its effect on a controlled release.

### 2.6. Characterization

#### 2.6.1. Morphological Properties

Structural and morphological characterization was performed using scanning electron microscopy (SEM, Hitachi, S-3400N-II, 20 kV), after solvent exchange and CO_2_ critical point drying. The samples were immersed in a solution of glutaraldehyde 2.5% (*w/w*) overnight, and then treated with ethanol solutions of increasing concentration (20, 30, 50, 70, 90 and 100% (*v/v*)), for 10 min each. The samples were dried using the CO_2_ critical point drying method, using an EMS K850 Critical Point Drier equipped with thermo-electronic heating and adiabatic cooling and temperature control of +5 °C for cooling and +35 °C during heating. For this, the samples were placed in the pressure chamber. This chamber was pre-cooled and immediately filled with liquid CO_2_ from a gas cylinder, which had a critical point at 31 °C and 1072 psi. It was heated to just above the critical temperature, reaching the critical pressure, at work conditions around 1500 psi and 35 °C. Finally, the samples were gold-covered via cathodic spraying.

The 3D matrix structure images obtained from SEM were processed using Diameter J, an open-source plug-in for image analysis software Image J. This tool segmented the SEM images into binary images, and it was possible to determine fiber and pore dimensions, using a methodology with defined criteria for the stabilization of the measured values average [27].

#### 2.6.2. 3D Computational Properties Optimization

To optimize the 3D matrix porous structure, a fiber 3D simulator was used. The dimensions and properties of the fibers, such as fiber length/width ratio, fiber wall thickness, fiber lumen, fiber flexibility and number of layers in the thickness direction, were used as input parameters in this 3D simulator, to produce the resulting 3D structure made from these fibers [28]. The simulation studies provided information about porosity and pore dimension and distribution on 3D matrix for one thousand simulated structures. The results were organized in regression and decision trees and several structures were selected to be produced, having the desired porosity and pore size distribution. This regular pore distribution contributed to obtaining an optimized 3D matrix for a more effective delivery system. Computational studies were performed using MATLAB^®^ (R 2020a, 9.8.0.1323502, MathWorks, Natick, MA, USA).

#### 2.6.3. Chemical Properties

The energy dispersion X-ray spectroscopy (EDX) method, coupled to the SEM detector, was used for the chemical characterization of the samples at an elementary level.

Fourier-transform infrared spectroscopy with attenuated total reflectance (FTIR-ATR, Thermo-Nicolet IS10, Waltham, Massachusetts, USA) was performed with 32 scans, a resolution of 4 cm^−1^ and a wavelength range of 600 to 4000 cm^−1^.

#### 2.6.4. Release Properties

Differential scanning calorimetry (Netzsch, DSC-204, Selb, Bavaria) was used to study the release of tea tree essential oil and microalgae *Chlorella vulgaris* components. The aluminum crucibles with samples were placed under an inert atmosphere of nitrogen in a temperature range of 18 to 150 °C and a velocity of 2 °C/min. For the studies, 18–27 mg of the sample was used. The differences between the sample crucible mass was quantified.

## 3. Results and Discussion

### 3.1. Structural and Morphological Properties

**Figure 1:**
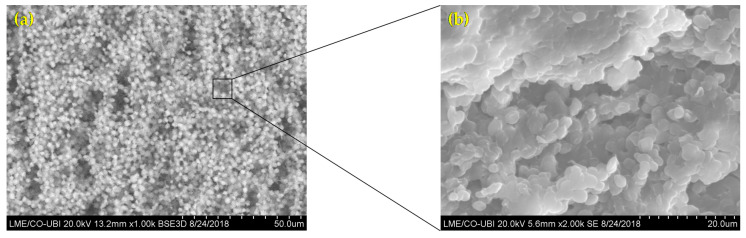
Scanning electron microscopy (SEM) images of *Chlorella vulgaris* morphology and structure at (**a**) 1000× and (**b**) 2000×.

The morphological features of the microalgae and MFC-NFC-CMC matrix materials were characterized using SEM. Figure 1 shows the structure of the microalgae *Chlorella vulgaris* sample. These microalgae presented as a freshwater unicellular and were spherical, with a cell size between 1 and 3 µm in diameter. These obtained diameters are due to the conditions used to produce *Chlorella vulgaris*. Cell sizes are increased when culture conditions are induced, such as light intensity and glucose [29,30].

**Figure 2:**
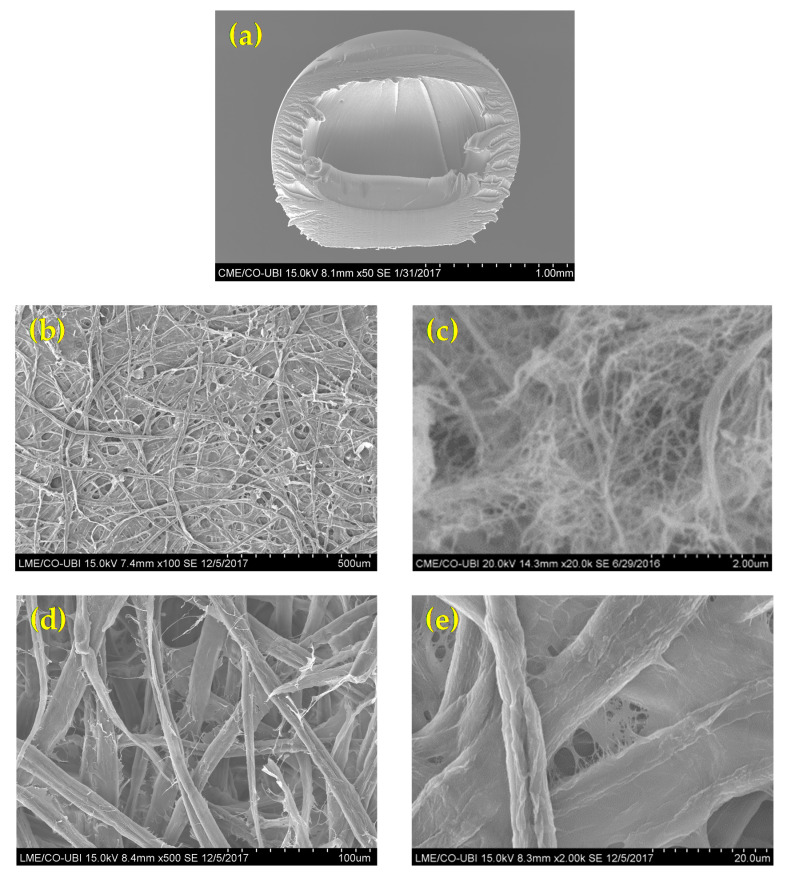
SEM images of morphology and structure of (**a**) delivery system cross-section; (**b**) microfibrillated cellulose (MFC) 3D matrix; (**c**) nanofibrillated cellulose (NFC) 3D matrix; (**d**) MFC-NFC-carboxymethylcellulose (CMC) 3D matrix at 500× and (**e**) 2000×.

Figure 2 showed the structure of the MFC-NFC-CMC 3D matrix sample. Morphological differences in the network were observed. It was also possible to visualize the spherical shape of the delivery systems (Figure 2a). These systems were cut, using a new, single-use blade in order to evaluate their internal matrix. The alginate, together with the divalent ions of the CaCl_2_ solution, formed a cross-linked structure where the molecules are physically trapped inside the matrix, able to migrate to the surrounding environment. For this reason, alginate as a crosslinking agent has often been used to produce DDS. The alginate gelation, through dripping in a solution containing Ca^2+^ ions, allows the formation of spherical DDS with regular shapes and sizes and a smooth surface, which can delay the release of the incorporated active substances [31]. The MFC matrix structure (Figure 2b) presented a uniform, intercalated, open and porous matrix, in microscale. The NFC matrix structure (Figure 2c) presented as a biomaterial with a porous, multi-structured 3D matrix, in the nanoscale. The combination of MFC and NFC allowed the production of a multi-structured hydrogel, capable of retaining and releasing therapeutic molecules. The combination of these biomaterials with CMC additive (Figure 2d,e) also allowed the inter-fiber bonding, with a more closed structure. Our previous studies have allowed us to conclude that the addition of CMC in nanocellulose matrices modifies its hydrophilicity, resulting in DDS with greater affinity for water, as it has more OH groups available for its interaction and intermolecular interactions with the surrounding environment [16]. For this purpose, we also used CMC as an additive in order to increase the affinity with water and control the release of therapeutic molecules from DDS. For the design of an innovative material used as an internal 3D matrix in DDS, we propose this combination of cellulose-based 3D structures, where the porosity and pore distribution has been controlled to obtain the desired release of molecules.

According to image analysis using the DiameterJ tool, the surface of the 3D porous matrix of MFC-NFC-CMC showed a porosity of 34%, approximately. The width of the matrix fibers showed a wide range from 0.0685 to 9.8 µm. The area of matrix pores showed a distribution between 0.0260 and 494 µm^2^. All cellulosic biomaterials present fiber and pore measurements in micro and nanoscale, allowing them to be multi-structured with great stability. This range of pore dimension and distribution is ideal for obtaining a biomaterial capable of incorporating biomolecules and releasing them in a controlled way to have the desired therapeutic effect. Since biomolecules are on a molecular scale, they are able to be released in a controlled way through a 3D porous matrix with uniform pore dimension and distribution at micro and nano scale, in order to have a therapeutic effect over time. The goal was to produce a biopolymeric delivery system with the best combination of the structural properties of the 3D matrix, such as porosity and uniform pore dimension and distribution. The pore dimension and distribution depend on the fiber dimension and the modification processes to which they are subjected [32], such as refining and high-pressure homogenization.

**Figure 3:**
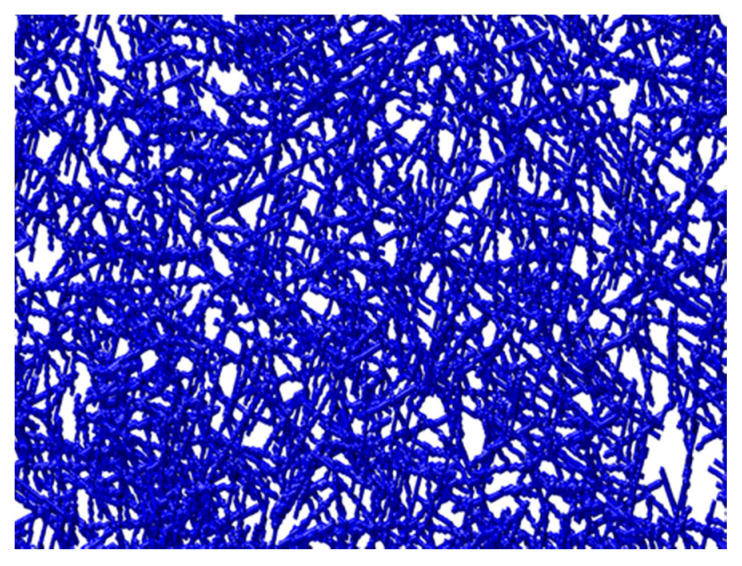
Example of 3D biopolymeric matrix obtained through the 3D fibrous-based computational simulator.

The integration of fiber data obtained from image analysis was performed to produce the 3D structure in a computational simulator, with a good experimental approach (Figure 3). The fibers were represented as chains of voxels in a 3D discrete spatial grid, using for computational simulations properties such as fiber length/width ratio, fiber wall thickness, fiber lumen, fiber flexibility and number of layers in the thickness direction, in order to produce a 3D structure made from modeled fibers [28]. The computational results were validated by comparing the surface porosity of the images obtained by the simulator, using the same image analysis methodology. The computational matrix showed a surface porosity of 36%, approximately. As this result is similar to that obtained experimentally, the computational methodology can be validated and used to optimize the structural properties of porous biomaterials computationally. The computational modeling and porosity optimization of three-dimensional structures are fundamental in the delivery systems application due to the influence they have on the release kinetics of therapeutic molecules [16]. The porosity of the biomaterials is crucial to achieve the desired release kinetics. It is essential to obtain an ideal polymeric delivery system with optimized porosities without compromising the 3D matrix structure.

The 3D polymeric matrix hydrogel was produced through stirring, pressure, temperature and time processes. These factors are decisive for producing a more or less porous biomaterial, since the process conditions are crucial to produce a material for different desired applications. Therefore, the process optimization also requires different conditions for each case. As the goal of this work was to obtain a porous system with the capacity to release therapeutic biomolecules in a controlled way over time, specific conditions were essential to obtain a 3D polymeric matrix with optimized porosity and pore dimension and distribution. For this purpose, computational models proved to be tools with great potential to improve the efficiency of DDS, as they were able to predict its cellulose-based 3D structure, without the need for extensive laboratory experiments. From the analysis of computational simulation results organized in regression and decision trees, it was found that the best combination of porosity (39.8%) and pore dimension and uniformity suited (0.02–120 µm^2^) to develop optimized biopolymeric delivery systems is achieved with the production of the biopolymeric matrix of MFC-NFC-CMC with agitation (500 r.p.m., using an impeller to ensure an effective mass transfer), pressure (vacuum filtration), temperature (20 °C) and time (100 min) controlled conditions. The DDS system consisting of an optimized 3D polymeric matrix and a crosslinking agent allowed the incorporation of biomolecules and their release due to the structure porosity optimization. The combination of experimental and computational methodologies was essential to design the necessary conditions to obtain a 3D matrix with crucial properties to be used in delivery systems for therapeutic molecules, such as essential oils.

### 3.2. Chemical Properties

**Figure 4:**
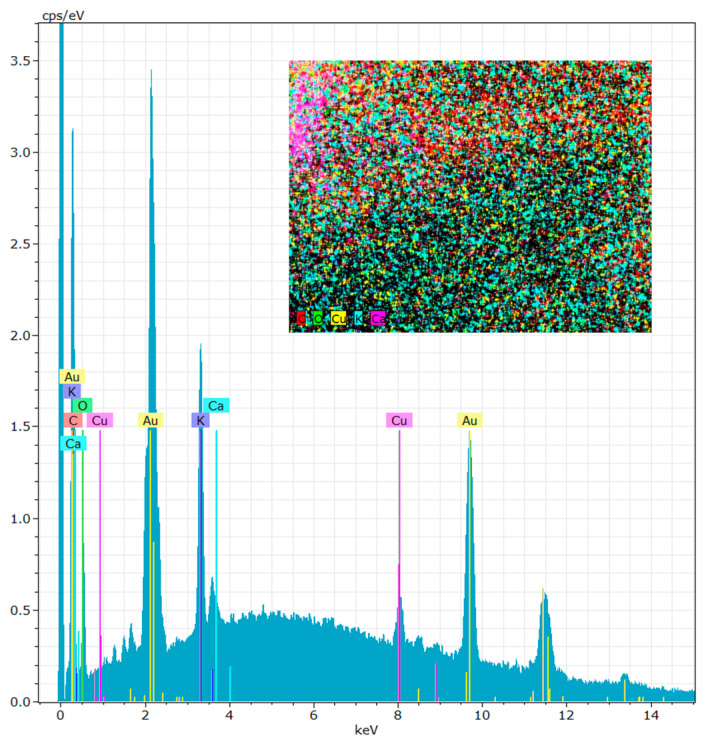
Energy-dispersive X-ray spectroscopy (EDX) spectrum of microalgae *Chlorella vulgaris.*

The EDX technique was performed to identify the chemical elements present in the microalgae *Chlorella vulgaris* structure after growth in culture medium (Figure 4). The results illustrated that the chemical elements present in greater amounts in the microalgae structure were carbon (66.47 ± 1.35%), oxygen (24.89 ± 0.86%), potassium (6.59 ± 0.14%), copper (1.43 ± 0.09%) and calcium (0.61 ± 0.06%). Most of these elements are considered essential mineral salts to have a therapeutic and cosmetic effect [33]. The presence of mineral salts in algae for cosmetic purposes is essential since the heat retention capacity for several hours is greater, simulating the blood circulation and, consequently, cleaning the skin of dead epidermal cells [33,34]. Figure 4 shows the presence of gold, but this element is not in the algae internal structure. The presence of gold is due to the experimental method carried out to visualize the samples in the SEM.

**Figure 5:**
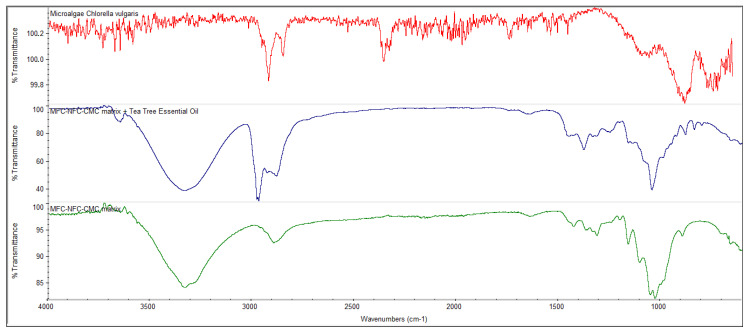
Fourier-transform infrared spectroscopy (FTIR) spectrum of microalgae *Chlorella vulgaris* (red) and MFC-NFC-CMC 3D matrix with tea tree essential oil (blue) and without tea tree essential oil (green).

The FTIR-ATR technique allowed to chemically characterize, identify and quantify the different biomaterials used in the DDS production, in order to evaluate whether the incorporation of biomolecules in the systems was effective. The FTIR technique also allowed the existence of physical retentions to be evaluated, without new bonding and without chemical reaction between the different components incorporated. Figure 5 shows the FTIR spectrum of the microalgae *Chlorella vulgaris* and the MFC-NFC-CMC 3D matrix with and without tea tree essential oil. The EDX results were also confirmed with the FTIR spectrum of the microalgae. The main bands to be identified in the microalgae FTIR spectrum are lipids (around 1740 cm^−1^), amides (around 1540–1660 cm^−1^) and carbohydrates (900–1200 cm^−1^) [35,36]. All these bands were identified in the microalgae *Chlorella vulgaris* FTIR spectrum. For the 3D matrix, an O-H stretching absorption at 3335 cm^−1^ was observed. This band is due not only to the OH groups present in the 3D biopolymeric matrix but also to the OH group present in the terpinen-4-ol structure, the active substance of the tea tree essential oil. The bands at 2971 and 1647 cm^−1^ are attributed to the stretching vibration of C–H in CH_3_ and stretching of the carboxylic acid group (C=O and C–O), respectively, of the 3D matrix and tea tree essential oil. These bands are not as pronounced in the spectrum of the 3D polymeric matrix without the essential oil. However, the presence of the C=O and C–O band also may be due to the oxidative treatment mediated by TEMPO as part of the NFC production process. Other characteristics of cellulose were also observed in the FTIR spectrums, such as angular deformation of C–H groups at 1379 cm^−1^, angular deformation of primary alcohol C–O bonds at 1251 cm^−1^, the absorption band of C–O–C bonds at 1045 cm^−1^ and β-glycosidic bonds between glucose units at 880 cm^−1^.

### 3.3. Release Properties

**Figure 6:**
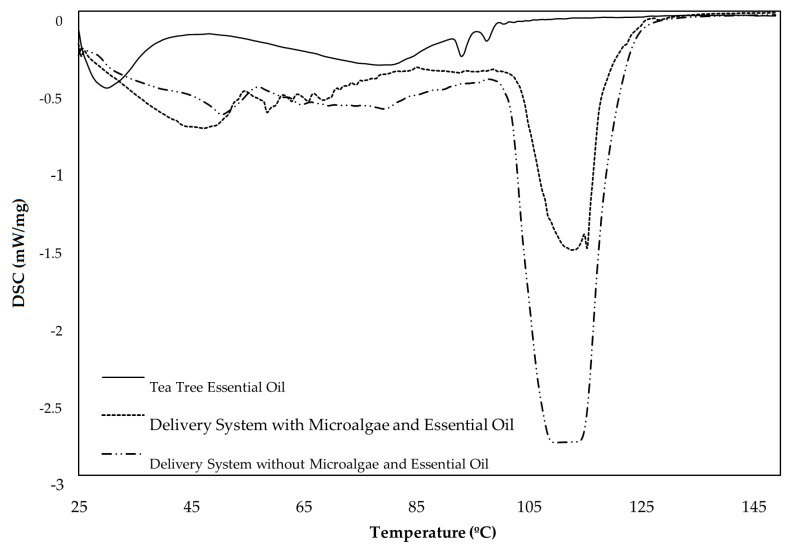
Comparison of DSC of the delivery systems containing the polymeric matrix and the tea tree essential oil, with and without microalgae *Chlorella vulgaris*, and the tea tree essential oil alone, which was not incorporated in the polymeric 3D matrix.

In addition to characterizing cellulosic materials, the DSC technique allows all phase transitions of biomolecules to be analyzed, which occurs during a temperature scan [37]. Thermal analysis methods can be used to study different materials, as well as to develop and prove the cosmetic product quality, in order to characterize cosmetics and their ingredients [38]. The use of DSC to understand biomolecule release studies was innovative, providing information about the performance of the delivery systems, characterizing their fusion behavior, obtaining a better understanding of their complex structure and also differentiating the different DDS. The DSC technique was also used to evaluate the evaporation process of the biomolecules incorporated in the DDS. The temperature increase allowed the energy absorbed to be determined using the samples, which is an advantage for the characterization of the release conditions of the therapeutic molecules, and how they can have a prolonged effect over the exposure time. DSC can be considered a useful tool to design DDS for cosmetic applications, determining the cooperativity of mixed biomaterials and biomolecules and revealing the interest in applying a system with a multi-structured 3D polymeric matrix. In this study, DSC was used to understand the release of tea tree therapeutic molecules and microalgae mineral salts when incorporated into biopolymeric delivery systems for cosmetic and dermal applications. The analysis of biomolecule releases was performed through the endothermic peaks in the heating curves. There is a release of the therapeutic molecules, which leads to a change in the baseline of the spectrum that is caused by the change in mass of the sample throughout the assay. As a result of this effect, the DDS remains without biomolecules. Therefore, a phase transition occurs in the DDS structures. These effects characterize the material very well. Figure 6 shows the DSC spectrum of biopolymeric delivery systems with tea tree essential oil and with or without microalgae *Chlorella vulgaris* and the tea tree essential oil. There is a difference between the peaks for three samples analyzed, showing the intermolecular forces between molecules. These forces can be strong or weak depending on the interaction between the different molecules [37]. It was possible to prove that the tea tree essential oil is quite volatile, being released between 25 and 38 °C. The tea tree was completely vaporized since in the beginning of the assay 18 mg of essential oil was used and, at the end, there were no components left in crucibles. Its incorporation in a system that allows its controlled release in a prolonged treatment is important for the molecules’ therapeutic effect to be effective. For the delivery system with microalgae and tea tree, endothermic peaks between 58 and 66 °C were observed. These peaks must correspond to the mineral salts released by the *Chlorella vulgaris* since they are not visible in the delivery systems without microalgae DSC spectrum. The mineral salts in this system may be able to retain the heat absorbed at this temperature ratio, presenting an effective cosmetic effect when used dermally [33]. The profiles of both delivery systems showed similarities in the endothermic peaks at approximately 50 °C. These peaks may correspond to the essential oil vaporization. Compared to the tea tree profile, the release temperature was later, corresponding to a more controlled and prolonged release when incorporated into a delivery system. Additionally, at a temperature above 100 °C, the evolution of the delivery systems was verified since from this point the components of the cellulose-based 3D matrix were released. The reactions that occur for cellulose samples at these temperatures are endothermic, attributed mainly to the water removal during heating [39]. The DDS evolution between the temperature range evaluated was also acceded by the difference in the crucible mass of the samples. At the beginning of the tests, DDS samples of about 25.3 ± 2.5 mg were used, and at the end, these samples presented a mass of 6.4 ± 1.1 mg, confirming that there was a controlled release of biomolecules and 3D matrix components from DDS. As the degradation rate depends on the stability of the biomaterials [37] used in each delivery system, the peaks were more or less intense, due to the presence or not of the microalgae *Chlorella vulgaris*. The DSC technique was essential to analyze each delivery system separately and what occurs in the retention of the cellulose-based 3D matrix. This technique also made it possible to study the retention of essential oil and microalgae in the 3D matrix within biopolymeric delivery systems. Therefore, the DSC technique allowed the release of the biomolecules to be quantified by increasing the temperature and also allowed the effect of association and retention that occurs in the combination of the various biomaterials in the 3D biopolymeric matrix to be evaluated.

The DSC curves provide information about the tea tree essential oil and its release from the polymeric matrix. The results indicate that the tea tree essential oil molecules are volatilized, changing phases, from liquid to vapor, in the range of 25 °C to 38 °C, when the oil is not inside the polymeric matrix. Considering that the skin reference temperature is 37 °C, these constitute an important limitation, because the time and amount of tea tree essential oil in direct contact with the skin when it is applied directly, without the polymeric matrix, is very low when compared with the essential oil inside the DDS polymeric matrix. The DSC curve also indicates that the incorporation of this essential oil in a delivery system with a 3D matrix of micro/nano and functionalized cellulose (MFC-NFC-CMC) retards the release of the essential oil molecules. For both DDS with and without microalgae, at 37 °C, the majority of the essential oil molecules are inside the polymeric matrix, augmenting the exposure time with the skin, therefore prolonging and improving the desired therapeutic effect. The development of an optimized 3D biopolymeric matrix that controls the release of the active molecules present in the tea tree essential oil consists of a competitive benefit for dermic systems. The incorporation of microalgae in these systems contributes to the controlled release of the algae minerals during the time of exposure. The DSC results indicate that after the water release and vaporization, which starts at 100 °C, the delivery systems are stable, and the remaining system consists of the nano/micro cellulose and alginate biopolymeric matrix, in concordance with the mass determination before and after the DSC analysis. The analysis of the DSC results also shows that there are no structural phase changes in this temperature range, in accordance with the absence of new chemical groups detected using the FTIR spectrum. This is indicative of the stability of cellulose-based biopolymers in the DDS. In the development of DDS formulations, chemical reactions can occur between active molecules and biopolymers, which is why it is important to identify the stability of the delivery system over a period of time and a range of temperature [40]. Therefore, the results indicate the stability and integrity of the nano/micro alginate delivery systems.

## 4. Conclusions

In this study, a delivery system with a 3D matrix of MFC-NFC-CMC capable of retaining and releasing therapeutic molecules from microalgae *Chlorella vulgaris* and tea tree essential oil was designed and developed. The 3D biopolymeric matrix proved to be a good biomaterial for the retention of functionalized biomolecules. Computational tools allowed the porosity and the pore dimension and distribution of the 3D matrix to be optimized, through the correlation of the ideal conditions to obtain this matrix. The delivery system developed with microalgae and tea tree allowed the controlled release of mineral salts and therapeutic molecules, such as terpinen-4-ol. Tea tree essential oil has very volatile constituents, being released quickly when it is not incorporated in a biopolymeric delivery system. It was possible to design and develop a biopolymeric delivery system containing a 3D cellulose-based matrix with the ability to maintain its integrity and stability over time and temperature and to incorporate, retain and release therapeutic biomolecules in a controlled way.

The present approach provided an innovative methodology for cosmetic and dermal applications, presenting a synergistic effect between the therapeutic biomolecules of microalgae *Chlorella vulgaris* and tea tree essential oil. Further studies should be carried out to investigate the functionalities of these cellulose biomaterials in other skincare applications, such as tissue masks.
